# Comparing Treatment Outcomes of Ampicillin-Sulbactam, Other β-Lactams, and Vancomycin in Blood Culture-Negative Infective Endocarditis

**DOI:** 10.3390/antibiotics10121476

**Published:** 2021-12-01

**Authors:** Se Ju Lee, Jung Ho Kim, Hi Jae Lee, Ki Hyun Lee, Eun Hwa Lee, Yae Jee Baek, Jin Nam Kim, Jin Young Ahn, Su Jin Jeong, Nam Su Ku, Seung Hyun Lee, Jun Yong Choi, Joon Sup Yeom, Young Goo Song

**Affiliations:** 1 Department of Internal Medicine, Severance Hospital, Yonsei University College of Medicine, Seoul 03722, Korea; playit@yuhs.ac (S.J.L.); QETU1111@yuhs.ac (J.H.K.); 0171S@yuhs.ac (K.H.L.); ESTHERLEE713@yuhs.ac (E.H.L.); STELLANGELA.BAEK@yuhs.ac (Y.J.B.); JAM764@yuhs.ac (J.N.K.); COMEBACKTOSEA@yuhs.ac (J.Y.A.); JSJ@yuhs.ac (S.J.J.); SERAN@yuhs.ac (J.Y.C.); JOONSUP.YEOM@yuhs.ac (J.S.Y.); IMFELL@yuhs.ac (Y.G.S.); 2 Division of Cardiovascular Surgery, Severance Cardiovascular Hospital, Yonsei University College of Medicine, Seoul 03722, Korea; HYMYHI@yuhs.ac

**Keywords:** antibiotics, blood culture-negative infective endocarditis, mortality, risk factors

## Abstract

Selection of proper antibiotics for blood culture-negative infective endocarditis (BCNIE) is difficult due to limited data on antibiotic regimens for BCNIE in existing literature. The aim of this study was to compare ampicillin-sulbactam, other β-lactams antibiotics, and vancomycin among patients with BCNIE to determine the proper antibiotic regimens. This retrospective study included adult patients with BCNIE admitted to Severance Hospital from November 2005 to August 2017. Patients were classified into three groups as, treated with ampicillin-sulbactam, other β-lactams, and vancomycin. The primary outcome was 1-year all-cause mortality. A total of 74 cases with BCNIE were enrolled in this study. There were no statistically significant differences in clinical characteristics between the three groups. One-year mortality did not significantly differ between the study groups either. Further, in-hospital mortality, 28-day mortality and overall mortality showed no difference. However, Cox-regression analysis showed nosocomial infective endocarditis as an independent risk factor and a protective effect of surgery on 1-year mortality. This study showed no clear difference in the outcomes of BCNIE as per the antibiotic therapy but suggested the beneficial effect of surgical treatment. With increasing global concern of antimicrobial resistance, it might be reasonable to select ampicillin-sulbactam-based antibiotic therapy while actively considering surgical treatment in BCNIE.

## 1. Introduction

Despite significant improvements in the management of infective endocarditis (IE), it is still associated with a high mortality rate [1]. For successful treatment of IE, administration of proper antibiotics to eradicate the implicating microorganisms is important [2]. Several guidelines have recommended antibiotic regimens and duration of therapy according to the causative pathogens [2,3,4]. Therefore, pathogen identification is essential for determining the treatment strategy of IE and not merely for the diagnosis.

However, as per a previous study, blood culture-negative infective endocarditis (BCNIE) accounts for 31% of all cases of IE [5]. A negative result of microbial blood culture in IE may result from administration of antibiotics before performing blood culture, infection caused by fastidious bacteria or fungi, and/or inadequate microbiological techniques [6,7]. There were concerns in the past that negative blood cultures might be associated with a delayed diagnosis and a worse clinical outcome, but recent studies have contradicted this theory [8,9,10].

In the case of BCNIE, clinicians take into consideration all the likely pathogens based on the patient’s status and local epidemiologic data. Two major guidelines on the management of IE have described several considerations for the choice of empirical antibiotic therapy in IE [2,3]. The Korean guideline for diagnosis and treatment of cardiovascular infections recommends ampicillin-sulbactam-based therapy for BCNIE and vancomycin-based therapy for patients unable to tolerate β-lactam antibiotics [11]. Nevertheless, selection of proper antibiotics in cases of BCNIE is difficult as the available data on suitable antibiotic regimens is scanty.

Accordingly, the choice of antibiotics for management of BCNIE is varied among physicians. The objective of this study was to compare treatment outcomes of ampicillin-sulbactam, other β-lactam antibiotics, and vancomycin among patients with BCNIE in a tertiary care hospital in South Korea to determine the proper antibiotic treatment of BCNIE. We also sought to identify other factors associated with treatment outcomes of BCNIE.

## 2. Results

**Figure 1:**
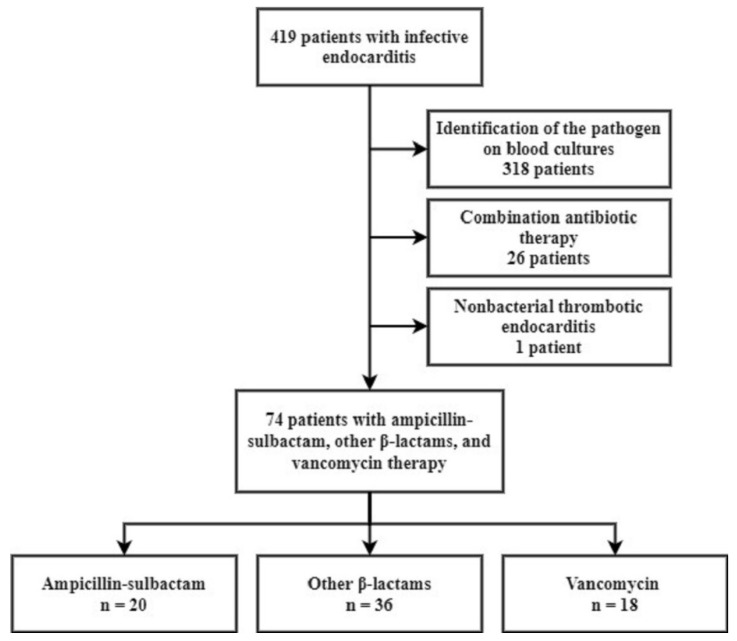
Flow diagram of the patients with blood culture-negative infective endocarditis.

A total of 419 patients with IE were identified during the study period. Of these patients, 345 patients were excluded for the following reasons: 318 patients were excluded due to identification of the causative pathogen on blood cultures, 26 patients were excluded as they received combination antibiotic therapy and 1 patient diagnosed with nonbacterial thrombotic endocarditis during follow-up was also excluded from the analysis (Figure 1). Finally, 20 patients in the ampicillin-sulbactam group (27.0%), 36 patients in the other β-lactams group (48.6%) and 18 patients in the vancomycin group (24.3%) were analysed.

### 2.1. Patient Characteristics

Seventy-four cases were finally selected with median age of 54.5 years. Of these 55.4% were male, 77.0% cases were of community-acquired IE and 87.7% were native valve endocarditis (NVE). Two patients with prosthetic valve endocarditis (PVE) had received valve replacement within 1 year of diagnosis. There was no statistically significant difference in clinical characteristics including valve status and Charlson Comorbidity Index between the three groups (Table 1). The nosocomial IE and community-acquired IE rates did not differ significantly among the three groups. The SOFA score for assessing severity also revealed no significant differences. Most of the patients in ampicillin-sulbactam group were treated with gentamicin (90%). The other β-lactam group consisted of 13 patients on penicillin, 11 on ceftriaxone, 6 on nafcillin, 5 on piperacillin/tazobactam, and 1 on cefazolin.

### 2.2. Treatment Outcomes

Clinical outcomes of the three groups are summarized in Table 2. The duration of antibiotic therapy was not different between the three groups. About three-quarters of the BCNIE patients received surgical treatment and the surgery was similarly performed in all three groups with similar indications. Analysis of the complication rates of new-onset heart failure, new conduction abnormality, paravalvular complication, renal failure, central nervous system involvement, and systemic embolic event during the treatment of IE, did not show significant differences among the three groups either.

**Figure 2:**
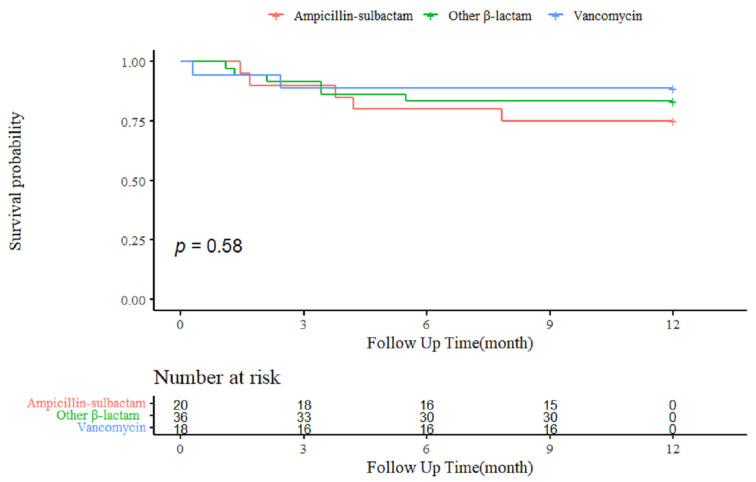
Kaplan-Meier curves for 1-year mortality rates of patients with BCNIE. *BCNIE* blood culture-negative infective endocarditis.

There were 13 cases of all-cause mortality during the 1-year follow-up. One-year mortality rate was 25.0% for ampicillin-sulbactam group, 16.7% for other β-lactams group, and 11.1% for vancomycin group, respectively and these results were not statistically significant (Table 2). Kaplan-Meier survival curve for 1-year mortality showed no significant differences between the study groups (Figure 2) (*p* = 0.58, log-rank). As secondary outcomes, in-hospital mortality and 28-day mortality also showed insignificant differences (Table 2). For overall mortality, 6 cases (30.0%) in ampicillin-sulbactam group, 7 cases (19.4%) in other β-lactams group, and 2 cases (11.1%) in vancomycin group were reported. Kaplan-Meier analysis for overall mortality also indicated no statistical differences between the three groups (*p* = 0.33, log-rank) (Figure S1). There was no significant difference in outcomes when comparing ampicillin-sulbactam group and the other antibiotic groups. (Tables S1 and S2).

### 2.3. Factors Associated with 1-Year Mortality of BCNIE

Multivariate cox analysis with backward elimination showed a higher risk of 1-year mortality among BCNIE patients in nosocomial infection cases (adjusted hazard ratio (HR) 6.14, CI 95% 1.41–26.69) and protective effect of surgical intervention (adjusted HR 0.18, CI 95% 0.04–0.79) (Table 3). Variable antibiotic treatments, such as gentamicin combination, ampicillin-sulbactam, other β-lactams, and vancomycin failed to show significant association with 1-year mortality.

## 3. Discussion

We compared clinical outcomes among patients with BCNIE treated with ampicillin-sulbactam, other β-lactam antibiotics, and vancomycin. In our study, clinical characteristics between the three groups were found to be comparable and the clinical outcomes in the three treatment groups did not show significant differences either. However, results of the Cox regression analyses revealed the beneficial effect of surgery on 1-year mortality.

Although scarce, there are a few studies in the existing literature on antibiotic selection in BCNIE. Werner et al. in their research, observed survival benefit with aminoglycoside therapy [12]. This study reported a mortality rate of 3% during treatment in the aminoglycoside group and 13% in the non-aminoglycoside group. Menu et al. showed the efficacy of standardized antimicrobial protocol for BCNIE by comparing the in-hospital mortality rates reported in the previous studies [13]. The standardized antimicrobial protocol tested by Menu et al. used amoxicillin with gentamicin for treatment of community acquired BCNIE and vancomycin with gentamicin for nosocomial BCNIE and post-surgical BCNIE. However, to the best of our knowledge there is no study at present, comparing the different antibiotic therapies in the management of BCNIE. Contrary to the study by Werner et al., gentamicin combination did not show significant association with 1-year mortality in the present study. Similarly, the different antibiotic groups had no significant effect on the 1-year mortality of the patients with BCNIE.

Recent decades have seen the rise in antimicrobial resistance become a global threat [14]. Antimicrobial resistance surveillance system for South Korea reported significant prevalence of antimicrobial-resistant bacteria in the environment [15]. Inappropriate antibiotics and prolonged use of broad-spectrum antimicrobial agents cause emergence of antimicrobial resistant species [16]. Regarding these aspects, ampicillin-sulbactam-based antibiotic treatment could be recommended as first-line therapy for patient with BCNIE as the ampicillin-sulbactam group showed similar outcome to the other groups in this study. Vancomycin could be reserved for alternative therapy when the patient is intolerant to β-lactam antibiotics. Besides, vancomycin is not always a safe antibiotic and may cause non-negligible adverse effects including nephrotoxicity and thrombocytopenia. Thus, avoiding unnecessary use of vancomycin is important.

*Staphylococcus aureus* remains the dominant pathogen in the epidemiology of IE and methicillin-resistant *Staphylococcus aureus* (MRSA) accounts for a sizeable portion of *S. aureus* worldwide. Consequently, clinicians treating patients with BCNIE frequently consider the possibility of MRSA being the causative pathogen out of caution and consider it apt to administer vancomycin during treatment. However, several studies have shown that the proportion of *S. aureus* in BCNIE is quite less (2–5%) and below the level for concern [5,17]. Considering the percentage of MRSA among *S. aureus*, the proportion of MRSA in BCNIE might be reduced further.

*Enterococcus* is also a dominant pathogen in the epidemiology of IE. As a vancomycin-based regimen is recommended for treating ampicillin-resistant enterococcal infective endocarditis, concern regarding ampicillin-resistant *Enterococcus* might prevent clinicians from selecting an ampicillin-sulbactam-based regimen. If regional epidemiological data indicate a high rate of ampicillin resistance in *Enterococcus*, using molecular methods such as Western immunoblotting might aid pathogen identification, and a vancomycin-based regimen would be a reasonable initial choice [18]. In this circumstance, vancomycin dose adjustment based on the area under the curve/minimum inhibitory concentration (AUC/MIC) ratio might improve treatment outcomes [19]. However, while 33 cases of enterococcal infective endocarditis (26 of *Enterococcus faecalis* and 7 of *Enterococcus faecium*) were confirmed in our institute during the study period, ampicillin resistance was confirmed in only one patient with *E*. *faecium* infective endocarditis. Additionally, in *E*. *faecalis*, which accounts for most enterococcal IE cases, the ampicillin resistance rate is low in South Korea [15,20]. These might explain why there was no difference in the outcomes between ampicillin-sulbactam and vancomycin groups in our study and supports the validity of ampicillin-sulbactam-based treatment as initial antibiotic therapy for BCNIE.

In addition, reducing unnecessary use of the other broad-spectrum β-lactam antibiotics is important keeping in mind the rise in antimicrobial resistance worldwide. Global dissemination of carbapenem-resistant Enterobacteriaceae (CRE) has been observed and exposure to carbapenem and cephalosporin were the most frequently mentioned risk factors associated with CRE acquisition [21]. Several studies have also implicated unchecked use of antibiotics such as carbapenem as the most common risk factor for the acquisition of multidrug-resistant *Acinetobacter baumannii* and *Pseudomonas aeruginosa*, which are another set of problematic hospital acquired pathogens [22]. In this view, the number of patients in vancomycin and other β-lactam group reminds us of the importance of antimicrobial stewardship and following standardized treatment protocol for the treatment of patients with BCNIE. Consultation with infectious disease specialists and close cooperation can reduce unnecessary antibiotics usage.

The protective effect of surgery for survival in patients with IE has been widely accepted by several authors [23,24,25]. Likewise, the benefit of surgical treatment in BCNIE has been suggested in a few studies. Lamas reported a 92% survival rate (33/36) for culture-negative NVE patients with surgery and 50% for the patients without surgery (3/6) [26]. Among 13 patients with culture-negative PVE who underwent surgery, 2 died of cardiac causes in the above-mentioned study. Menu et al., reported a 5.1% mortality rate in 177 BCNIE patients in their study, with a 59.9% rate of surgery and suggested the benefit of an aggressive surgical approach [13]. We demonstrated the protective effect of surgery in BCNIE patients conclusively by performing Cox regression analysis (adjusted HR 0.18, *p*-value 0.023) and thus, it can be stated that surgical treatment in BCNIE might be considered in the same manner as in IE.

Several studies reported poor outcome of nosocomial IE with reported mortality of 27–82% [27,28,29]. In our study, consistent with previous studies, nosocomial BCNIE (58.8%) showed a markedly higher 1-year mortality rate than community-acquired BCNIE (5.26%) and suggested an association with 1-year mortality in multivariate cox regression analysis (adjusted HR 6.14, *p*-value 0.015).

It is obvious, in case of BCNIE for clinicians to consider fastidious or intracellular pathogens as possible aetiologies. Several studies on BCNIE have shown a considerable proportion of these pathogens like *Coxiella burnetii* and Bartonella species [5,26,30]. However, IE caused by *C. burnetii* and Bartonella species have been rarely reported in South Korea [31,32]. In addition, lower prevalence of IE caused by fastidious or intracellular pathogens in our study population might be inferred from lower in-hospital mortality rate compared with the previous studies [17,33]. Therefore, we believe that the results of this study might be valid in the cases of BCNIE excluding non-culturable microorganisms by serologic assessment or molecular methods such as 16s rRNA sequencing.

This study has a few limitations. First, the majority of BCNIE cases in our study were that of NVE. Among the 9 PVE patients, 2 received valve replacement within 1 year. Therefore, the conclusion of our study might not be applicable to PVE patients. Second, as the epidemiology of IE may vary regionally, our conclusion based on single-institutional data may not be generally applicable. Still, we maintain our conclusion is sustainable regarding the main causative pathogens of IE are similar globally [24,34].Third, serologic assessment of non-culturable microorganisms like *C*. *burnetii* and *Bartonella* species was rarely performed in our study. Another limitation of our study is the relatively small sample size due to the inclusion of patients from a single-institution only. Thus, further studies with more patients are needed to clarify the choice of first line antibiotic therapy in BCNIE. However, given the low prevalence rates of MRSA and ampicillin-resistant enterococci, a similar conclusion would likely be reached with a larger study population. As mentioned above, MRSA is not common; *Enterococcus faecalis* accounts for more than 90% of cases of enterococcal infective endocarditis and its resistance rate to ampicillin is low, even in nosocomial infection [20,35]. Lastly, we cannot ignore that the results of our study are influenced by confounders because of the retrospective study design. Despite these limitations, this study is valuable as this is the first study to compare different antibiotic regimens in BCNIE.

## 4. Materials and Methods

### 4.1. Study Design and Patient Population

This retrospective study enrolled adult patients diagnosed with IE admitted to Severance Hospital, a large tertiary-care teaching hospital in South Korea, from November 2005 to August 2017. Patients were included if they met the following criteria: (1) Patients above 17 years; (2) diagnosed with IE and admitted to Severance Hospital; and (3) No isolated pathogen from either blood culture or valve tissue culture obtained from patients who underwent surgery. Patients were excluded if they met the following criteria: (1) Patients who received combination antibiotics therapy and (2) diagnosed with non-bacterial thrombotic endocarditis.

IE was defined as definite or possible according to the modified Duke criteria [36]. The institutional review board of Yonsei University Health System Clinical Trial Centre approved this study (4-2018-0248). Because the study was retrospective and the data were anonymized, the IRB waived the requirement for informed consent.

### 4.2. Antibiotic Groups

Patients were classified into three groups, based on the treatment received on the index date as: (a) treated with ampicillin-sulbactam, (b) treated with other β-lactams (penicillin, nafcillin, cefazolin, ceftriaxone, or piperacillin/tazobactam) and (c) treated with vancomycin. The index date was defined as the date when the first blood culture revealed no microbial growth. Ampicillin-sulbactam and vancomycin groups were assigned since they were recommended in the treatment guidelines. As there were many cases of administration of antibiotic therapy outside the guidelines, we defined a group as treated with other β-lactams, although this is the not recommended antibiotic therapy for BCNIE as per the guidelines but is administered for IE caused by specific pathogens. Patients who received a combination of antibiotics in each group were excluded from this study. Ampicillin/sulbactam was administered as a 3-g dose every 6 h, as recommended in the treatment guidelines. The dose was adjusted in patients with impaired renal function. Vancomycin was initially administered at a dose of 15–20 mg/kg every 12 h. The dose was adjusted in patients with impaired renal function; in half of the vancomycin group, dose adjustment was guided by therapeutic dose monitoring.

### 4.3. Variables and Definitions

The primary outcome was 1-year all-cause mortality. To analyse death outside the hospital and long-term survival, we used mortality data obtained from the Ministry of the Interior and Safety of South Korea, which collects death related information of all Korean citizens. Secondary outcomes included in-hospital all-cause mortality, 28-days all-cause mortality, and overall all-cause mortality. The date of diagnosis of IE was considered the starting point, from which the days until mortality were counted. Nosocomial infection was defined as an infection that occurred after 48 h of hospitalization without any evidence of infection at admission. Charlson Comorbidity Index was calculated at admission for classifying patients according to overall comorbidity [37]. The Sequential Organ Failure Assessment (SOFA) score was used to measure patients` severity of illness. Systemic embolic complications included pulmonary embolism, splenic infarction, coronary embolism, and peripheral limb embolism.

### 4.4. Statistical Analysis

Differences in patient characteristics and outcomes were assessed between the three groups using the chi-square test for categorical variables and one-way ANOVA or Kruskal-Wallis test for continuous variables. Continuous variables were checked for normal distribution by Shapiro-Wilk test. Chi-square test or Fisher exact test were used to find differences of categorical variables between ampicillin-sulbactam and other antibiotic groups. Survival analysis was performed using Kaplan-Meier curve and log-rank test to estimate long-term outcome. Adjusted Cox regression analyses were performed to assess the risk factors for 1-year mortality of BCNIE and the association between antibiotic therapy and 1-year mortality. Variables with *p* < 0.05 in univariate analyses were entered into backward stepwise multivariate Cox model. Hazard ratios and 95% confidence intervals were calculated based on multivariate model. A *p*-value of <0.05 was considered statistically significant. All statistical analyses were performed using R V.4.0.5 (The R Foundation for Statistical Computing, Vienna, Austria).

## 5. Conclusions

In conclusion, our study did not find a clear difference in treatment outcomes among the various antibiotic therapies used for BCNIE; however it suggested the potential advantage of surgical intervention for the management of this disease. Thus, in terms of global concern and increasing incidence of antimicrobial resistance, it might be reasonable to select ampicillin-sulbactam-based antibiotic therapy and actively consider surgical treatment in BCNIE, especially for community-acquired NVE.

## Figures and Tables

**Table 1:** Clinical characteristics of patients with BCNIE.

	Total (*n* = 74)	Ampicillin-Sulbactam (*n* = 20)	Other β-Lactams (*n* = 36)	Vancomycin (*n* = 18)	*p*-Value
Demographics					
Age, years, median (IQR)	54.5 (44.25–67.75)	54 (32.75–69.25)	54 (48.5–68)	55 (45.25–65.75)	0.908
Male sex (%)	41 (55.4)	9 (45.0)	23 (63.9)	9 (50.0)	0.343
Community acquired (%)	57 (77.0)	15 (75.0)	28 (77.8)	14 (77.8)	0.969
Nosocomial (%)	17 (23.0)	5 (25.0)	8 (22.2)	4 (22.2)	0.969
Valve Status (%)					0.189
Native valve	65 (87.7)	17 (85.0)	34 (94.4)	14 (77.8)	
Prosthetic valve	9 (12.2)	3 (15.0)	2 (5.6)	4 (22.2)	
Involved valve (%)					0.351
Aortic valve	31 (41.9)	9 (45.0)	17 (47.2)	5 (27.8)	0.373
Mitral valve	42 (56.8)	12 (60.0)	18 (50.0)	12 (66.7)	0.478
Tricuspid valve	5 (6.8)	1 (5.0)	3 (8.3)	1 (5.6)	0.869
Pulmonary valve	4 (5.4)	2 (10.0)	0	2 (11.1)	0.133
Multiple valves	7 (9.5)	4 (20.0)	1 (2.8)	2 (11.1)	0.104
Comorbidities (%)					
Previous IE	2 (2.7)	0 (0.0)	1 (2.8)	1 (5.6)	0.573
Predisposing valve condition	25 (33.8)	5 (25.0)	13 (36.1)	7 (38.9)	0.611
Patients with previous valve surgery or prosthesis	13 (17.6)	4 (20.0)	4 (11.1)	5 (27.8)	0.299
Patients with cardiac devices	3 (4.1)	0	1 (2.8)	2 (11.1)	0.192
Diabetes mellitus	13 (17.6)	1 (5.0)	10 (27.8)	2 (11.1)	0.071
Chronic heart failure	2 (2.7)	1 (5.0)	1 (2.8)	0 (0.0)	0.637
Renal disease	3 (4.1)	1 (5.0)	1 (2.8)	1 (5.6)	0.860
Liver disease	2 (2.7)	0	1 (2.8)	1 (5.6)	0.573
Solid cancer	9 (12.2)	4 (20.0)	4 (11.1)	1 (5.6)	0.382
Hematologic malignancy	2 (2.7)	0	2 (5.6)	0	0.338
Recent chemotherapy	5 (6.8)	3 (15.0)	2 (5.6)	0	0.170
Connective tissue disease	1 (1.4)	1 (5.0)	0	0	0.254
Immunosuppressive therapy	1 (1.4)	1 (5.0)	0	0	0.254
Charlson comorbidity index, median (IQR)	4 (0–4)	2 (0–3.25)	1 (0–3.25)	1 (0.25–2.75)	0.805 ^1^
SOFA score, median (IQR)	1 (1–2)	1 (1–2)	1 (1–2)	1 (1–2)	0.984 ^1^
Antibiotics use in the last 3 months (%)	11 (14.9)	2 (10.0)	6 (16.7)	3 (16.7)	0.774
Central venous access (%)	6 (8.1)	3 (15.0)	2 (5.6)	1 (5.6)	0.417
Modified Duke criteria (%)					0.507
Definite IE	29 (39.2)	9 (45.0)	15 (41.7)	5 (27.8)	
Possible IE	45 (60.8)	11 (55.0)	21 (58.3)	13 (72.2)	
Gentamicin combination (%)	54 (73.0)	18 (90.0)	24 (66.7)	12 (66.7)	0.133

BCNIE blood culture negative infective endocarditis, IQR interquartile range, IE Infective endocarditis, SOFA Sequential Organ Failure Assessment. ^1^ *p* values were calculated using Kruskal-Wallis test.

**Table 2:** Treatment outcomes in patients with BCNIE.

	Total (*n* = 74)	Ampicillin-Sulbactam (*n* = 20)	Other β-Lactams (*n* = 36)	Vancomycin (*n* = 18)	*p*-Value
Duration of antibiotics treatment, days, median (IQR)	30 (23.25–42.75)	31 (20.75–42)	30.5 (24.75–50)	28 (23.25–41.5)	0.670 ^1^
Surgery performed (%)Indication of surgery (%)	56 (75.7)	15 (75.0)	28 (77.8)	13 (72.2)	0.901
Congestive heart failure	53 (71.6)	15 (75.0)	26 (72.2)	12 (66.7)	0.845
Prevention of embolism	24 (32.4)	9 (45.0)	11 (30.6)	4 (22.2)	0.308
Paravalvular complications	8 (10.8)	2 (10.0)	4 (11.1)	2 (11.1)	0.991
Pacemaker infections	2 (2.7)	0	1 (2.8)	1 (5.6)	0.573
Uncontrolled infections	1 (1.4)	1 (5.0)	0	0	0.254
Clinical outcomes (%)					
New-onset heart failure	8 (10.8)	3 (15.0)	3 (8.3)	2 (11.1)	0.743
New conduction abnormality	8 (10.8)	4 (20.0)	2 (5.6)	2 (11.1)	0.249
Paravalvular complication	5 (6.8)	1 (5.0)	2 (5.6)	2 (11.1)	0.697
Renal failure	7 (9.5)	3 (15.0)	2 (5.6)	2 (11.1)	0.493
CNS involvement	19 (25.7)	6 (30.0)	10 (27.8)	3 (16.7)	0.593
Systemic embolism	6 (8.1)	2 (10.0)	2 (5.6)	2 (11.1)	0.730
Mortality (%)					
1-year mortality	13 (17.6)	5 (25.0)	6 (16.7)	2 (11.1)	0.522
In-hospital mortality	7 (9.5)	1 (5.0)	4 (11.1)	2 (11.1)	0.727
28-days mortality	4 (1.4)	0	3 (8.3)	1 (5.6)	0.417
Overall mortality	15 (20.3)	6 (30.0)	7 (19.4)	2 (11.1)	0.346

BCNIE blood culture negative infective endocarditis, IQR interquartile range, CNS Central nervous system. ^1^ *p*-value was calculated using Kruskal-Wallis test.

**Table 3:** Cox regression analysis for 1-year mortality.

	Univariate Analysis		Multivariate Analysis ^1^	
	Hazard Ratio (95% CI)	*p*-Value	Hazard Ratio (95% CI)	*p*-Value
Nosocomial IE	14 (3.9–52)	<0.001	6.14 (1.41–26.69)	0.015
Surgery performed	0.075 (0.02–0.27)	<0.001	0.18 (0.04–0.79)	0.023
SOFA score	1.6 (1.1–2.3)	0.008		
Charlson comorbidity index	1.2 (1.1–1.4)	<0.001		
Gentamicin combination	0.41 (0.14–1.2)	0.11		
Antibiotics groups				
Ampicillin-sulbactam	Reference			
Other β-lactams	0.66 (0.20–2.17)	0.498		
Vancomycin	0.44 (0.09–2.27)	0.326		

CI confidence interval, IE Infective endocarditis, SOFA Sequential Organ Failure Assessment. ^1^ Multivariate analysis with backward elimination.

## Data Availability

The data presented in this study are available on request from the corresponding author.

## Supplementary Materials

The following are available online at https://www.mdpi.com/article/10.3390/antibiotics10121476/s1, Table S1: Comparison of treatment outcomes between ampicillin-sulbactam and Other β-lactams group, Table S2: Comparison of treatment outcomes between ampicillin-sulbactam and vancomycin group, Figure S1: Kaplan-Meier curves for overall mortality rates of patients with BCNIE.
