# Supplementary material for: Comparing Treatment Outcomes of Ampicillin-Sulbactam, Other β-Lactams, and Vancomycin in Blood Culture-Negative Infective Endocarditis
Source: Antibiotics (Basel). 2021 Dec 1;10(12):1476. doi: 10.3390/antibiotics10121476 (PMC8698808; doi:10.3390/antibiotics10121476)
Supplement: Supplementary file 1 [file antibiotics-10-01476-s001.zip › antibiotics-1456984-supplementary.pdf]

Table S1. Comparison of treatment outcomes between ampicillin-sulbactam and Other β-lactams group

	Ampicillin-sulbactam (n=20)	Other β-lactams (n=36)	p value
Duration of antibiotics treatment, days, median (IQR)	31 (20.75-42)	30.5 (24.75-50)	0.51[1]
Surgery performed (%)	15 (75.0)	28 (77.8)	1
Clinical outcomes (%)			
New-onset heart failure	3 (15.0)	3 (8.3)	0.655[2]
New conduction abnormality	4 (20.0)	2 (5.6)	0.172[2]
Paravalvular complication	1 (5.0)	2 (5.6)	1.000[2]
Renal failure	3 (15.0)	2 (5.6)	0.336[2]
CNS involvement	6 (30.0)	10 (27.8)	1.000
Systemic embolism	2 (10.0)	2 (5.6)	0.611[2]
Mortality (%)			
1-year mortality	5 (25.0)	6 (16.7)	0.497[2]
In-hospital mortality	1 (5.0)	4 (11.1)	0.645[2]
28-days mortality	0	3 (8.3)	0.546[2]
Overall mortality	6 (30.0)	7 (19.4)	0.514[2]

IQR, interquartile range, CNS Central nervous system

[1] p value was calculated using Mann Whitney U test

[2] p values were calculated using fisher test

Table S2. Comparison of treatment outcomes between ampicillin-sulbactam and vancomycin group

	Ampicillin-sulbactam (n=20)	Vancomycin (n=18)	p value
Duration of antibiotics treatment, days, median (IQR)	31 (20.75-42)	28 (23.25-41.5)	1[1]
Surgery performed (%)	15 (75.0)	13 (72.2)	1
Clinical outcomes (%)			
New-onset heart failure	3 (15.0)	2 (11.1)	1[2]
New conduction abnormality	4 (20.0)	2 (11.1)	0.663[2]
Paravalvular complication	1 (5.0)	2 (11.1)	0.595[2]
Renal failure	3 (15.0)	2 (11.1)	1[2]
CNS involvement	6 (30.0)	3 (16.7)	0.454[2]
Systemic embolism	2 (10.0)	2 (11.1)	1[2]
Mortality (%)			
1-year mortality	5 (25.0)	2 (11.1)	0.41[2]
In-hospital mortality	1 (5.0)	2 (11.1)	0.595[2]
28-days mortality	0 (0.0)	1 (5.6)	0.474[2]
Overall mortality	6 (30.0)	2 (11.1)	0.238[2]

IQR, interquartile range, CNS Central nervous system

[1] p value was calculated using Mann Whitney U test

[2] p values were calculated using fisher test

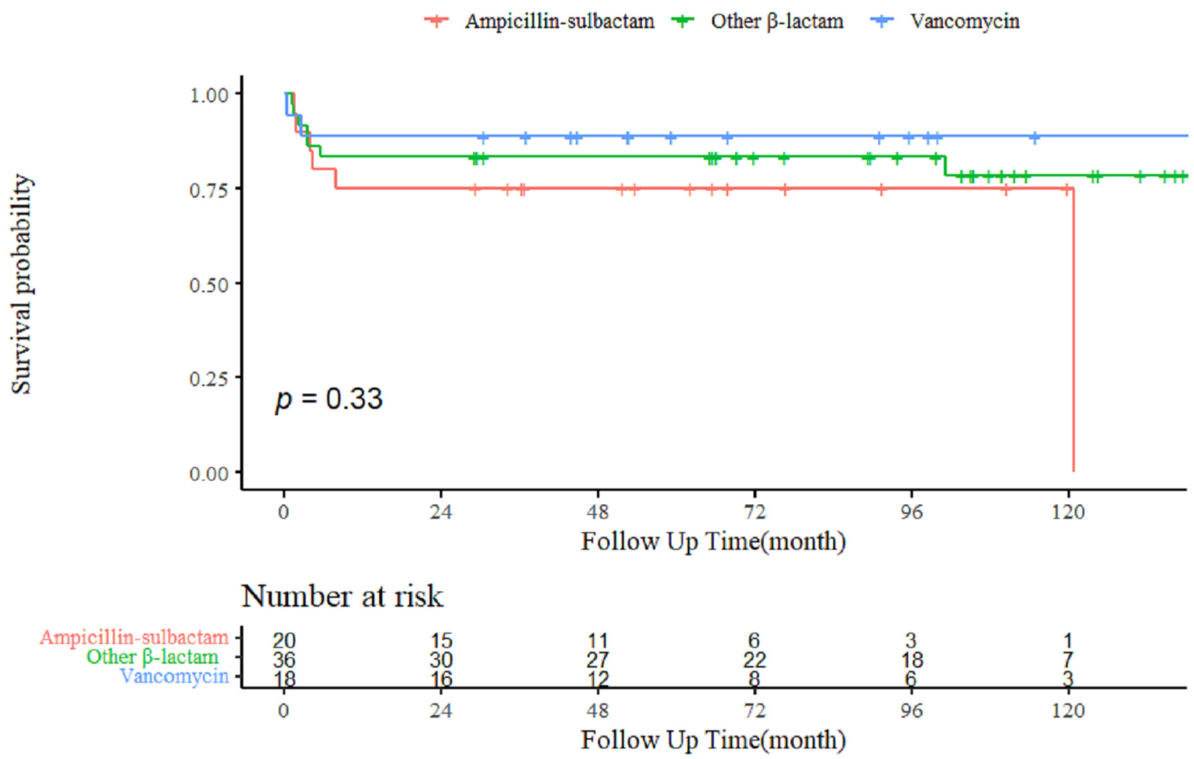

Figure S1. Kaplan-Meier curves for overall mortality rates of patients with BCNIE.
BCNIE blood culture-negative infective endocarditis
